# Why Are Scientists Not Providing More Leadership in the World?

**DOI:** 10.1093/function/zqac035

**Published:** 2022-07-06

**Authors:** Ole H Petersen

**Affiliations:** School of Biosciences, Cardiff University, Sir Martin Evans Building, Museum Avenue, Cardiff CF10 3AX, UK

I recently spoke at a conference on the future relationship between scientists in the UK and the European Union (EU). The theme was topical, as UK researchers are increasingly worried that they may not be able to participate in “Horizon Europe” (HE), the European Union's key funding program for research and innovation, with a budget of ∼100 billion US dollars. The EU and the UK had, at the end of 2020, agreed a Trade and Cooperation Agreement (TCA), which included the full participation of the UK in HE. However, the agreement on UK participation in HE has not been implemented, effectively being blocked by the EU because of the continuing political dispute concerning the so-called Northern Ireland protocol, which is part of the TCA. At the time of writing this editorial, the UK's Parliament is debating a new law that would allow the UK to break the TCA, an international law signed by both the UK and the EU. Understandably, this is unacceptable to the EU. Nevertheless, the political dispute over the Northern Ireland protocol has nothing to do the with the need for effective scientific collaboration in Europe.

There is also a political dispute between the EU and Switzerland, so Switzerland has also not been able to become associated with HE. This is not only a problem for the UK and Switzerland, but represents a significant weakening of European Science. Not least in the Biomedical Sciences, and certainly in Physiology, Switzerland and the UK have enormous strengths and are, according to most measures, the leading European nations.

In response to this threat to scientific excellence in Europe, a “Stick to Science” (“put science collaboration before politics”) campaign has been initiated. This movement represents >5000 researchers and research organizations across Europe and was initiated by the two top Swiss Universities (ETH Zürich [Swiss Federal Institute of Technology in Zürich] and EPFL [Swiss Federal Institute of Technology in Lausanne]), Universities UK, The Royal Society, and the Wellcome Trust. An open letter signed by the Presidents of these institutions was sent to the President of the European Commission, Ursula von der Leyen, on 22nd June 2022, asking her to intervene personally to save effective European science cooperation. These events were the backdrop to the conference mentioned above, at which I spoke on behalf of Academia Europaea.

During the discussion at the conference, which became rather broad, the general issue of why we (scientists) allow politicians to effectively “walk over us” came up. In response to a question, I said: “Unless Europe gets its act together, we will be relegated to second class. Maybe universities, which are quite powerful institutions, commanding significant resources, should take initiatives themselves and establish alliances that are not necessarily dependent on what their governments want.” In the specific example discussed above, one could envisage a situation in which an organization representing all major European Universities would refuse to participate in HE, as well as applying to their national funding bodies, unless universities in Switzerland and the UK were included in HE. If such solidarity were possible, it would be immensely powerful and the political leaders in the EU, the UK, and Switzerland would simply have to accept the scientific case for close cooperation.

No doubt, many will think that this is completely unrealistic and also undemocratic. However, parliamentary democracy is not necessarily the best way of regulating all affairs in society and there are many examples of issues that could be regarded as highly “political” that are actually decided by experts. In the UK, for example, the Bank of England's base rate, that influences all interest rates by private banks, was determined by the government until the Chancellor of the Exchequer (Finance Minister) in 1997 delegated this key decision—obviously of great importance for the national economy—to the Bank of England's Monetary Policy Committee. In this context, one could wonder why politicians still think it is reasonable that government ministers and their administrators should decide on school curricula, number of places for medical students at universities, and arrangements for running hospitals, just to mention a few examples from the UK. Would it not be much better if these issues were regulated by people who actually, via their education and practice, know something about these matters?

We are living in a world that during the last 50 years has undergone dramatic developments and transformations due to advances in science and their applications. However, in spite of scientists having created the frame for the way we now live, their voice is not prominent in public debates and therefore they have very little power. In contrast, the media pay enormous attention to politicians and the so-called celebrities, those who are famous for being famous! Thus, a politician, Al Gore—who is often credited with taking the initiative in creating the Internet—became much better known worldwide than the scientist, Tim Berners-Lee, who invented the World Wide Web. While the internet undoubtedly overall is beneficial to the world, and indeed is now indispensable for our daily lives, many serious problems are also emerging and scientists are not sufficiently influential in determining regulations.

During the Covid crisis, we have seen many governments claiming to “follow the science,” but in reality not doing so. Some medical experts have achieved a degree of prominence in the media, but pronouncements on the epidemiology, as well as vaccination programs, by politicians who have no expertise in these areas, have generally been much more widely distributed. The reason is of course that the politicians, in contrast to the medical experts, have real power.

The classical case of a clash between politicians and scientists is the development of the atomic bomb and the realization of the scientists who were responsible for its creation that they were powerless to prevent it from being used. The Pugwash conferences on science and world affairs, recipient of the 1995 Nobel Peace Prize, was an attempt by scientists to prevent the use of nuclear weapons and had at some point a degree of success (Non-Proliferation Treaty), but has ultimately been unable to prevent an increasing number of nations from developing such weapons. Just now, in the middle of Russia's war against Ukraine, the threat by Russia of using nuclear weapons is uppermost in our minds.

The great Russian physicist, dissident, and peace activist Andrei Sakharov correctly pointed out that we can only solve the many major problems in this world if we have an effective and powerful World Government. Unfortunately, we seem to be getting further and further away from this goal. However, it might be somewhat easier to create an international body representing scientists and technologists, who could influence the way we run our societies. This would be a long-term development that could, for example, be led by National Science Academies in combination with prominent Universities. Unfortunately, the International Science Council (formerly the International Council of Scientific Unions), which could in principle play such a role, has not, in my personal opinion (and based on my experiences as a former Secretary General of the International Union of Physiological Sciences—IUPS), been an effective and influential body. It has a much lower profile than several national academies, including the Royal Society, the National Academy of Sciences, and the German National Academy of Sciences Leopoldina. Physiologists could also do with a more powerful international voice, although IUPS has made some progress by the establishment in 2021 of its Academy of Physiology, which may have the potential to become an influential body, if utilized properly.

In general, universities have much larger resources at their disposal than scientific societies and alliances between these very different types of organizations, which have many common interests, would give considerable additional strength. Academia Europaea, the European Academy of Sciences and Humanities, has shown the way by the establishment of a number of knowledge hubs in various European Universities, funded by these institutions. This has broadened the base of the academy and provided much needed additional manpower and resource. However, such schemes need to be expanded to operate on a much larger scale.

Meanwhile, it would be good for scientists and their institutions to be less timid in their reactions to what governments tell them to do. To be effective, this requires strong solidarity between, for example, Universities, who all too often feel that they are in competition with each other for resources and therefore are looking to gain advantages for themselves at the expense of others. However, at least with regard to the still dominant national funding, solidarity between groups of universities in different countries should not be problematic as they would not be competing for funding from the same sources. In any case, “United we stand, divided we fall.”

## Funding

None declared.

